# The Uncertain Presence: Experiences of Living with Metastatic Breast Cancer

**DOI:** 10.1080/01459740.2021.1941003

**Published:** 2021-06-30

**Authors:** Cinzia Greco

**Affiliations:** Wellcome Trust Research Fellow, Centre for the History of Science, Technology and Medicine, University of Manchester, Manchester, UK

**Keywords:** United Kingdom, France, metastatic breast cancer, crisis of the presence, uncertainty

## Abstract

Drawing from interviews with women with metastatic breast cancer in the UK and France, in this article I analyze uncertainties linked to this condition. In particular, I show how the impossibility of foreseeing the evolution of the condition, also as an indirect consequence of medical innovation, initiates an irreparable disruption of life after diagnosis. I further show how the lives of the patients are not only limited by the illness, but also by the difficulty of finding a place in society. I argue that such experiences are best understood through the concept of the *crisis of the presence.*

In this article, I analyze how the experiences and subjectivities of women with metastatic breast cancer (MBC) in the UK and France are characterized by complex uncertainties that can cause life disruptions that are often impossible to mend. Such uncertainties are caused by the fact that the survival time for MBC is extremely variable. This situation has been exacerbated by the innovations in treatments, the hopes attached to treatments, and the complexities of navigating the therapeutic options. In this analysis, I offer a new understanding of long-term conditions, in particular, the cases in which the indeterminacy of the condition makes it impossible to close the disruption. I propose the use of the concept of the crisis of the presence as a way to conceptualize long-term conditions as both embodied and located in society.

Starting in the 1970s, there has been rising attention in the social sciences of medicine on the effect of long-term chronic diseases (Bury 2010). One of the most influential approaches to the study of debilitating and progressive diseases is that of the "biographical disruption." Bury (1982) described the disruption as involving, first, the body and the difficulty of living one's life as before; second, the biography of the ill person and their conception of self; and, third, the mobilization of resources and responses to cope with the disease.

Most criticisms of the concept of biographical disruption have focused on the fact that it does not consider cases in which there is no single disruption to identify, or in which the person who is ill does not live their illness as a disruption (see the syntheses in Bury 2010; Williams 2000). Disability studies scholars have further argued that the issue is not in the chronically ill body, but in a social organization that creates disability by excluding those with nonstandard capacities and those not functional to the capitalist economy (Oliver 1996). In this article, I focus however on the tendency to present the biographical disruption as part of a trajectory that is always closed by the composure of the crisis through specific resources. I do not deny that some patients are able to mobilize resources and find new social roles and places in the world, but I argue that in cases such as that of MBC, in which the evolution of the disease is unforeseeable, the crisis cannot be really closed. The problem, therefore, is that we lack theoretical tools to conceptualize crises that do not end. The concept of biographical disruption has been applied in previous studies of MBC (Kenne Sarenmalm et al. 2009; Lewis et al. 2016) in which researchers recognize the difficulties experienced by the patients; however, these studies focus strongly on building trajectories in which the disruption is closed. This means that disruptions that do not end, which I have found in the cases of my interviewees, still remain understudied in the case of MBC.

Alternative concepts proposed to analyze the uncertainty of patients with a severe diagnosis include precariousness and risk. Medical anthropologists have mostly used the concept of precariousness to identify not so much the illness itself, but the social conditions, such as the economic and social hardship that can cause and be caused by health conditions, creating a vicious circle (cf. Manderson and Warren 2016). Only recently has the concept of biosocial precarity been advanced to describe conditions that can cause health problems as well as stigma, social marginality, and legal problems, such as the use of illicit substances in pregnancy (Premkumar et al. 2020). However, the concept of precariousness seems less adequate to describe the social and moral disorientation that illness brings, even when it is not strongly linked to social marginality and economic difficulties.

The concept of risk has been used more widely, including for breast cancer. Studies have shown that risk in breast cancer is linked to uncertainties in defining malignant lesions (Löwy 2007), as well as the ambiguities of statistical risk (Jain 2013). Breast cancer risk further extends to healthy women through family predisposition and BRCA1 and 2 genes diagnoses (Gibbon 2007). Women who have successfully completed treatments for early-stage breast cancer also remain at risk, as the possibility of relapse and the sequelae of the treatments mean that there is no return to normality, and that the whole process from diagnosis to the end of the treatments could be considered a biographical disruption (cf. Trusson et al. 2016). Risk becomes even more problematic and complex in the case of advanced cancer: patients with advanced cancer enrolled in clinical trials have difficulties conceiving the future development of their illness (Brown and de Graaf 2013). This also applies to MBC, because having the diagnosis means that one will almost certainly die of breast cancer. However, as I discuss in this article, the survival range for MBC is particularly broad – a few months or several years, with a median of three years (cf. Gobbini et al. 2018; Mariotto et al. 2017). Paradoxically, this makes planning the future even more complex: rather than the moment of the diagnosis, it is the whole experience of the illness and its treatments, including the uncertainties involved, that could be conceived as a "crisis of the presence."

The combination of an uncertain risk and other physical and social limits deriving from the disease makes the crisis of the presence an alternative and heuristically rich concept. The notion of "crisis of the presence" was formulated by the Italian anthropologist De Martino (1973[1948], 1982[1959], 2000[1958]) to describe the experience of losing oneself and one's place in the world as a consequence of negative experiences that greatly limit one's agency, especially in challenging contexts characterized by poverty and deprivation (De Martino 1982[1959]). The crisis of the presence allows us to theorize subjectivity as a body–society relation. The presence is both the sense of the self and capacity for agency, and it is embodied and affected by both the social context and crises, such as illness or grief. The crisis of the presence has some point of contact with the concept of the “loss of self” used to describe the experience of chronic illness (Charmaz 1983). However, while the loss of self is characterized by an interactionist focus on self-*image* and loss of status and face, the concept proposed by De Martino focuses on the existential dimension of the loss of one's place in the world. I consider here subjectivity as the interaction of De Martino's concepts of the presence – sense of the self and capacity for agency; and of place in the world – the social roles available to the person. While based on earlier philosophical concepts, such definition is coherent with the more recent anthropological theories of subjectivity as in becoming and inseparable from its biosocial and political environment (e.g. Biehl and Locke 2017).

In his initial formulation De Martino considered the crisis of the presence to be limited to magic-using societies (De Martino 1973[1948]); however, in his later work he recognized that all societies are open to the contradictions that bring about crises of the presence (De Martino 2000[1958]: 15–21). Two of his main works (De Martino 1982[1959], 2000[1958]) are based on ethnographic research with peasant women in Basilicata, southern Italy, in the 1950s. These positioned crises of the presence in a broader context of poverty, endangerment by poor harvests, high infant mortality (De Martino 1982[1959]: 78–79) and gendered subalternity in which becoming widowed was particularly dire (De Martino 2000[1958]: 86–87). Subsequent work has used the concept of crisis of the presence to describe chronic disease, including the difficulty of giving it a meaning and the creation of a second subjectivity characterized by the illness, and has also linked it to the concept of biographic disruption (Peláez-Ballestas et al. 2013). The crisis of the presence has been used to describe illness narratives among women in Samnium (also in southern Italy) as redemptive rituals, in which problems deriving from gendered inequality are embodied as physical symptoms (Pandolfi 1990, 1993). Honkasalo's work in Northern Karelia in Finland linked the concept to the region's increased depopulation and marginalization, showing how the crisis emerges in illness (2009) and is exacerbated by the condition of being part of a religious minority (2015). Beneduce (2005) has argued that biomedicine has not solved the crises of the presence linked to illness and that modernity has not removed the economic precarity forming the broader context of the crises of presence.

I argue here that the crisis of the presence can show and describe aspects that are outside the remit of biographical disruption. The crisis of the presence is particularly useful in analyzing the illness experience in which, as a consequence of a diagnosis that brings indeterminacy and uncertainty, there is no way to recompose the disruption of self. This is the case of MBC, despite – and, to some degree, also because of – the medical innovation in the treatment of the condition. Furthermore, the crisis of the presence allows us to understand the disruption brought by the illness as simultaneously located in the body and in the social context. In particular, as the ethnographic applications of the concept have underlined, the crisis of the presence allows us to look at a larger social dimension of the crisis, avoiding the biographical disruption approach's often unbalanced focus on individual patients and their personal networks. De Martino's observation about the "critical moments that are intrinsic to capitalistic civilization as such" (De Martino 2000[1958]: 36) continues to be relevant also to our current condition of hyper-capitalism. Previous forms of capitalism also offered limited space for people with any kind of disability, but hyper-capitalism exacerbates ableism – the discrimination of those not conforming to idealized sets of abilities – with its deregulation, welfare cuts, and compulsory push to self-entrepreneurship (Goodley 2014).

In the following, I first offer an overview of the therapeutic options available for MBC and the role of the organization of health care in the UK and France. After presenting a summary of my research methods, I show how the complexity of the landscape of treatments available creates hopes about the efficacy of therapeutic options, but also difficulties in navigating them. I also show how difficult it is for patients to foresee the possible evolution of their condition in absence of certain knowledge, and how their lives are restricted, not only by the condition, but also by the lack of social accommodation for their needs.

## Metastatic breast cancer in the United Kingdom and France

The metastatic phase of breast cancer is when cancer spreads to other organs, usually leading to the death of the patient. Early-stage breast cancer is considered curable, whereas MBC is increasingly defined as treatable but not curable – a definition which emphasizes that the patient will likely die of cancer, but that it is possible to keep the situation under control for some time. Breast cancer is often presented as an example of the progress in cancer treatments. It is one of the few cancers for which the treatments allow long-term (more than five years) survival for most patients (cf. Timmermann 2013). Epidemiological studies have shown an improvement in median survival time for MBC starting in the 1990s, attributing this phenomenon to the new drugs that have been introduced since then, including hormonal treatments and monoclonal antibodies (Andre et al. 2004; Sundquist et al. 2017).

The most important drugs for MBC target either estrogen receptor-positive breast cancer or cancers with an overexpression of the protein HER2. Tamoxifen, which is used to treat estrogen receptor-positive breast cancer, is often considered the first example of a targeted therapy for cancer (Jordan 2006). There is an increasing number of MBC drugs approved and on the market, and others still in the experimental phase and accessible through clinical trials. The rising importance of clinical trials with limited accessibility also complicates and makes therapeutic pathways uncertain. The MBC tumors that do not react to treatments targeting estrogen, progesterone, or HER2 receptors are defined as *triple negative* (cf. Keating et al. 2016), and do not currently have targeted therapies available; however, several clinical trials are exploring new treatments for these tumors.

The therapeutic landscape for patients with MBC is further complicated and made unstable by the organization of health care systems. The two national health care systems I analyze here are both advanced and inclusive, covering the vast majority of the population and with public institutions providing high levels of professional care. The British healthcare system is free at the point of use for those resident in the UK. The National Health Service (NHS), however, has seen the gradual introduction of an internal market since the Thatcher-led Conservative government (1979–1990), which aimed to reduce the role of the state to that of a purchaser, decentralizing both decision-making and provision, and introducing competition (see Klein 2013). The French system is based on national health insurance, with most of the costs that patients incur either directly covered or subsequently refunded by a public insurance system. The different organizations of health care indirectly affect the availability of treatments. Newly introduced cancer treatments are extremely costly, and the French and British health care systems have different ways to manage these costs. In France, the focus is on negotiating the price with the pharmaceutical industry, and on establishing different levels of public coverage according to the efficacy of the drug. In the UK, where the NHS entirely covers most treatments, the National Institute for Health and Care Excellence (NICE) establishes whether the cost–result ratio justifies the introduction of the drug in the NHS (Abecassis and Coutinet 2017). Since 2010 there is a specific government fund, the Cancer Drug Fund, exclusively dedicated to cover the cost of drugs not yet approved by NICE. However, in some cases British patients can lack access to drugs that are at least in part covered by the French health care system.

## Method

This analysis derives from a comparison of experiences of patients with MBC in the UK and France. Between late 2017 and 2018, I conducted research focused on MBC's recent history over the last 40 years, as represented in medical literature and perceived by medical professionals and MBC patients. In the UK, I conducted 26 interviews, 16 with medical professionals and 10 with patients with MBC. The interviewees were contacted through medical institutions and patients' associations. I focus my attention here on the part of the research involving the patients. The in-depth interviews with them were organized around the exploration of the illness experience the patients had before the breast cancer diagnoses; the differences between the primary and secondary diagnosis, when the interviewee was not metastatic at first diagnosis; their relationship with medical professionals; and the impact of MBC on everyday life. The interview approach was open-ended, with patients often exploring additional themes, and interviews ranged from 30 minutes to three hours. I became interested in the experiences of patients with MBC while conducting research in the Île-de-France region between 2012 and 2014. This earlier research comprised 58 interviews with patients and 26 with medical professionals and focused on breast cancer surgery in general. Here also the contact was through medical institutions and patients' associations, and in part through a snowballing approach. I collected extensive illness narratives with patients. Three of them, who were living with MBC at the moment of the interview, offered detailed accounts that included all the aspects I explored more explicitly in the interviews in the UK.

There was a brief interval between the two studies, and the sample size is different, but it is valuable to analyze the two sets of data together. The UK and France both have highly advanced and inclusive healthcare systems; thus, comparing illness experiences in these two countries shows that the crisis of the presence is not linked to the shortcomings of a specific healthcare system, nor to healthcare deprivation. This allows us a focus on how crises of the presence are linked to how uncertainty in the biomedical context is configured in hyper-capitalism.

The overall group was diverse in terms of years since secondary diagnosis, severity of metastases, and level of activism in associations. However, minorities and women of lower socio-economic class are under-represented: most of the interviewees were middle class; all were white, and only one (among the UK cohort) had parents with an experience of migration. This can probably be explained in part by the fact that contact had been made mainly through patients' associations, in which only certain profiles of patients tend to be involved, and in part by a degree of self-selection among the patients who answered to requests for interviews. The ages of the interviewees ranged from mid-30s to the late-70s and the time since MBC diagnosis from a few months to ten years. While the interviews in France were all conducted in the Paris metropolitan area, some UK interviews also took place in medium and small towns. Most interviews were at the interviewees' homes, with some conducted in other places such as cafes, according to the preference of each interviewee. All the names I use are pseudonyms, and some minor details are changed to avoid identification. I based the comparative analysis on the extended case method (Burawoy 1991), which focuses on linking the ethnographic data with structural aspects and on individuating in what the data collected deviated from existing theories. The extended case method was particularly useful in identifying how the experiences of patients with MBC suggest the need to revise existing theories on long-term illnesses. Although narratives are constructions dependent on culturally available narrative structures (Garro and Mattingly 2000), located in a specific time, and cover only part of the overall experience of a disease, they are an important and rich point of access to patients' illness experiences. Narratives can illuminate the contradictions and open-ended questions raised by an MBC diagnosis. I conducted a pooled case comparison (West and Oldfather 1995); that is, although the two research projects were conducted at separate times and with partially different aims, I have reanalyzed the materials using a common interpretative scheme. This involved returning to the interviews looking for common themes (including themes that were originally not central for the research conducted in France) rather than merely comparing the results of the two research projects.

## Treatment hope and the promises of oncology

Since the 1990s, the number of therapies available for MBC has increased. The new drugs and the intense research activity linked to innovation in breast cancer engage the patients in forms of treatment hope. Delvecchio Good (2001) observed how medical innovation engenders economies of hope, through which both doctors and patients build expectations about the efficacy of new treatments. The patients I interviewed were aware that their condition is not curable, but had reason to hope that biomedicine could control the progression of the disease. Florence, a French patient in her late forties at the time of the interview who had a relapse and metastatic diagnosis a decade earlier, described her therapeutic pathway as follows:

[For some months, I have been taking] Afinitor [….] I am doing a scan next week, and if the lungs have recovered well, I will take it again because, for the tumour, it has worked well, the PET scan was really good. [In the past, I had] chemo, I had rays, and I also had luck that my metastases have been treated with radio-frequencies in the vertebrae. It is still rare, and I really had luck [to meet] this doctor who loves everything new, who's passionate, and wants his patients to profit from everything new.

At the time of the interview, Florence was taking a monoclonal antibody that had been introduced recently. The drug, despite its collateral effects, on the respiratory system in particular, was providing good results. Florence had received other innovative therapies before, including radio frequency ablation – otherwise used mostly for liver lesions (cf. Crocetti et al. 2010) – which was used to remove her bone lesions. Florence appreciated the fact that her oncologist allowed her to access new therapies that lengthened her life beyond the three years the statistics mention and that could potentially control her disease even longer. Treatment hope is important for women who are undergoing innovative therapies, as well as those for whom such therapies are only a future option. This is the case of Holly, a British woman who was diagnosed in her late thirties and, at the time of the interview, was taking tamoxifen with good results:

I take a tablet at night and I am pretty much fine. I feel so lucky. I live such a normal life [….] The average life expectancy is three years, so I have had two of those years already, does that mean that I'm going to die next year? But I feel so well…. I know [that] I am on my first drug, and there is a lot more hormone-therapies and chemotherapies that I have to try. So logically, I should have years left […] and because I responded so well to tamoxifen, I got a greater chance.

Holly felt in good health and had difficulties in matching her health condition with the statistical data that seemed to leave her with only a year. Moreover, she knew that there were other drugs if tamoxifen stopped working. Holly's hope to live for several more years – marked by the term "logically" – seems reasonable, and in any case, is perfectly in line with biomedical discourse. The difficulty in anticipating the results of the therapies in individual cases (cf. Brown and de Graaf 2013), along with the difficulties in accessing the most experimental (and costly) therapies, makes the therapeutic pathways of other patients much more fragile and open to a crisis of presence. Kathy, a woman in her fifties, had a metastatic diagnosis with involvement of the lungs, liver, and bones. Her tumor was HER2 positive, and this was immediately presented as good news in a difficult situation, as her oncologist estimated that Herceptin could control the progression of the cancer. In Kathy's case, however, the benefits were limited, and the drug was not providing the results the medical professionals were hoping for. Together with her oncologist, Kathy decided to try a new drug, Kadcyla, which was not offered at that time by the NHS in the area where she was living. She ended up funding the treatment herself. Although the cost for a fully out-of-pocket treatment was thousands of pounds, she happened to have the sum available at that time. Despite the material and moral resources invested in the new treatment, the results were not encouraging: the drug was not working, and she was "running out of options." Kathy had resigned herself to undergoing another round of chemotherapy, but was also aware that that perspective did not offer her much hope:

I thought "I [will go through] another chemotherapy", but I knew that, as you move along the chemotherapy line, it's a sort of diminishing return. You know they are less effective, so I wasn't hopeful. [But my consultant] found me a clinical trial, and I thought it was a good one for me, and it was the last place on the trial, the last slot. And I said, "I'll take that", nothing to lose, and I started fairly quickly on it and [it was] a miracle – within 10 days my cough is gone. I felt completely different; it was just astonishing. I couldn't quite believe it. I remember thinking "this can't be happening".

The clinical trial that Kathy entered (this time without out-of-pocket expenses) because she had "nothing to lose" was the event that finally brought her a significant improvement. The drug limited the development of the disease, and when I interviewed Kathy in 2018, she told me she was one of the few patients still in the trial, as most of the participants had died. However, the therapy seemed effective for Kathy and allowed her an active life with limited side effects.

Every treatment is intertwined with the hope that it can extend a patient's life, and that if a specific treatment does not work, others can be used. However, finding an effective treatment often requires a complex search, in which the social context and the cultural and economic capital of the patient play an important role.

Even when there are significant personal resources to invest, there is uncertainty linked to the complexity, and often the difficulty, of accessing treatments. The therapies do not allow patients to close the crisis brought on by the diagnosis. The crisis is kept open by the need to look for and gain access to new treatments, as well as by the fact that therapies may not work for an individual patient, as Kathy's experience with Kadcyla shows. Treatments dominate patients' present, while the future is marked by the impossibility of knowing how the disease will progress, as I discuss in the next section.

## The unforeseeable future and the difficulty of (not) knowing

As mentioned, the median survival time for patients with MBC is around three years, with subgroups of patients living longer (see Sundquist et al. 2017). At the time of the diagnosis, it is not possible to foresee how the disease will develop and what life expectancy a patient might have – a lack of control over the future similar to the experience described as "temporal anomie" by Olson (2011). This uncertainty takes over the women's lives, profoundly altering also their present. The medical establishment is, in many cases, incapable of giving answers and information to the patients. This was the case for Nicole, a French patient who was 50 years old when she was told that her cancer had come back and that she had lesions in her bones. During the interview, she told me how difficult it was to obtain information about the evolution of her disease: "It's impossible to have statistics," she said. Nicole would have liked more information on life expectancy for her subtype of cancer, on the likelihood of being able to return to work, and whether it was indicated for her to remain in her apartment, situated on the third floor of a building without an elevator. The replies to her precise questions were evasive and unsatisfying: "They say to me: 'it's impossible to know; you may end hit by a car when you get outside the hospital.' These are replies way off the mark (*des réponses à coté de la plaque*)." Frustrated by the evasiveness of the medical staff, Nicole booked an appointment with another oncologist in another hospital in Ile-de-France:

I got an appointment in another hospital. I arrived with my dossier, and I immediately told him, "I have no intention of changing hospitals nor to become your patient. On the other hand, I would just like to find some[one] who answers my questions". He was lovely and answered, he took 45 minutes to tell me "but no, it isn't worth it to move houses [because bones could break spontaneously] in 25% of the cases, so this leaves you with 75% of the cases, and moreover it is not necessarily the leg, you could break an arm, and moreover, we will put you [in] a plaster cast, you will be fixed, and you will climb your floors".

Nicole needed to have reliable information that could help her manage the impact of the disease on her everyday life, and the consultation with the new doctor was helpful in this regard. Other women had similar difficulties, and the lack of knowledge about the evolution of their condition in the near future left them stuck also in their present. The incapacity of biomedicine to give adequate answers to patients has already been noted by Bury (1982) in terms of its inability to explain the causes and meaning of a disease. In the case of MBC, this is exacerbated by the high variability of the survival time. The impossibility of anticipating the development of the disease means that a moment of recomposing the crisis, in which the patient might live with a limiting but stable condition, becomes elusive. Moreover, this uncertainty limits social spaces the patients can find in their present. Understanding MBC as a crisis of the presence allows us to see how the disease does not bring the patients to take on a new social space, even a limiting one, but rather takes away their points of reference, such as life expectancy, that could allow them to find a new place in society. In De Martino's terms, what is impossible is "being in history [which] means giving a formal horizon to suffering, objectifying it in a particular form of cultural coherence" (De Martino 2000[1958]: 17).

In this situation, as Robyn's story shows, it is very difficult to adjust to their post-diagnosis life. At the time of the interview, Robyn, a British patient, was about to start a new treatment, but it was impossible to know whether it would work. She said that, if the treatment did not work, she could be "on [a] very short-time life span," but, she continued, "my consultant, actually he has got a number of patients who respond really well on capecitabine." This uncertainty about the future affected her present:

[I don't know] whether I am better off just quitting work and taking medical retirement. And that's great, I can go and afford to have two years off, if I'm lucky enough to stay well, effectively living on the salary I am on now and not working. But do I do that on the basis that the stats tell me [that] I'll probably be dead in the period, which is a horrible way to think, but I have to think like that, or do I not do that because I might be one of the exceptional responders who is still here in 15 years, and actually that means that I can't afford to do that? There are really hard decisions to make.

In contrast to Nicole who was looking for statistics and practical information that could help her to take decisions about her life, Robyn seemed to have this information, but the information itself was somewhat conflicting. The statistics stated that the median survival time is three years, but the clinical experience of her oncologist showed many patients living longer due to therapeutic progress. Robyn did not know what her future situation would be, and the ambiguity of the future was blocking her present. The variety of treatments available and their uncertain outcomes introduced a new form of temporality that set the time of the women's lives with the temporality of the periodic scans because, as Kathy said to me, "You can't plan ahead because you don't know what is gonna happen to the next scans."

In this situation, many patients tried to extend the value of the present, such as Daisy, a British woman in her sixties, who told me how she and her daughters decided to use part of the money she had put aside to travel together and build good memories for the future. Other women tried to use the present to project their presence in the future, such as Kathy, who said that, immediately after the diagnosis, she started to put together memory boxes and write letters for her children as a way to be there for them after her death. For women with small children, there is little practical support available to help them with their parental duties, while trying to take care of themselves. As already shown in previous research (Bell and Ristovski-Slijepcevic 2011), the experience of motherhood suffers a temporal disturbance. Women are pressured on one hand to maintain a normal life for their children, and on the other hand, they want to share as many good moments as possible with their children because they know that there might not be other opportunities in the future. It is also important to underline that the disease not only unsettles relationships with the children, but also with partners, relatives, and friends (cf. also Kenne Sarenmalm et al. 2009; Lewis et al. 2016). Letitia, a British patient in her early seventies, who had been living with MBC for several years, spoke during the interview about her divorce: "I always say that my having had cancer had something do to with that [the divorce]. Because […] my husband was always one for making plans, always going for new things, and [after the diagnosis] I became more hesitant about long-term planning."

The experiences presented suggest that a deep uncertainty about the future characterizes even the best moments, influencing patients' psychological well-being and their views on their future and personal relationships.

## “A tiny life”: the impact of metastatic breast cancer on social life

The women I met were aware they would be on treatment for the rest of their lives, and that the diagnosis triggers a number of changes, some immediate, others developing over time (cf. also Bury 1982; Charmaz 1983). Many women told me how they ceased or significantly reduced driving, and how this obviously limited their mobility, independence, and social life. Many women also saw their work life interrupted or deeply limited (cf. also Lewis et al. 2016: 1175). The pain and debilitation brought on by the illness and collateral effects of the treatments forced many to reduce their professional activities or stop working entirely. Nicole was one of these cases, and described her life a few months after the diagnosis: "I do a weekly chemo since August. I stopped working […] and it's probable that I will not work anymore, my life has been seriously shaken (*bouleversée*). Here you see me on my feet; I manage to do some things, but still it's not a normal life."

Nicole told me that quitting work was not her choice: she loved working and found her job very gratifying, but her physical condition did not allow her to follow the rhythms of the work. Despite these difficulties, Nicole tried to adapt to her new life and find other activities that could be accessible with her health condition. Abby, a British patient in her fifties, shared how the illness had cascading effects on her life. Prior to diagnosis, she had an active and rich life. She spent many years abroad with her family and had always helped her husband with his work, but she said that her life had completely changed after the diagnosis. She used to be self-employed, but the work she was able to do was significantly reduced at the time of the interview. Due to the disease and the side effects of the treatments, she had difficulties walking, and, while describing the impact of the diagnosis in her everyday life, she said: "I feel like [my life] has shrunk, it was very expansive before and now, I mean, it's just tiny, I barely walk up the road, and I have to get a taxi for almost everywhere I go."

Robyn also shared that the illness had made her world and her professional possibilities more limited:

I travelled with my partner. We have gone to [South-East Asia], and we worked as data collectors […], but I can't do that anymore because no one would insure me anymore, and that's really hard. I mean life has changed overnight just because you got… I mean, I am well right now, what would stop me to go to America? But I wouldn't get insured […], and that's really frustrating […]. I mean, it feels like doors are closing, that's what I find really hard. I'm only 45.

In this passage, Robyn referred to the fact that her life changed "overnight" and that, even in the moments in which her health condition was stable, the illness had reduced her opportunities because it introduced an ineliminable risk factor in her life. The social management of disease, as a risk to contain, added to Robyn's physical condition by closing doors in her life. Similar to Nicole and Abby, Mary, another British patient also in her fifties, had to leave her job and struggled to adapt to post-diagnosis life:

The biggest thing for me is the fact that you don't feel valued anymore because our world values us by the definition of our job. I am not talking about my whole life, [but] out there, we are who we are in our jobs. Sadly, that's the type of world we live in… all this knowledge [that I have of] my profession, it's wasted [because] I am not in that arena anymore, and it's hard. It's not about my ego; it's about the value to society, to the world, and feeling that you are making a contribution. I feel now that I just look at the world. I watch others rather than participate.

Mary's words conveyed deep pain and a difficulty to live in a world in which some points of reference that were important for defining her identity had disappeared. The lives of the women I met had unexpectedly collapsed on themselves. All of them were active and dynamic before the diagnosis, but their space for action had significantly reduced. The feeling described by Mary, to be a spectator rather than the protagonist in one's life, was common also among other interviewees. Some of the women I met were able to create new social roles linked to their condition, for example as cancer activists (cf. Gibbon 2006). However, also those who took on a role of this kind were explicit in underlining the limitations that the disease caused in their lives. These difficulties were partly linked to the illness, and partly to the ways in which society reacts to the illness.

Disability studies have pointed out that part of the difficulties that disabled people suffer are caused not by their physical impairment, but by the fact that society is incapable or unwilling to include people with different needs and abilities (Oliver 1996). These considerations are also confirmed by the experience of the women with MBC. Many of the women I met would have preferred to continue working, but quitting was the only option because the organization of work does not accommodate the needs linked to the illness experiences, such as those of patients with MBC. Robyn's experience is significant in this regard. It was the diagnosis, itself, even before the physical consequences, that limited her life. In a social context in which the working life is an important component of one's identity, as Mary underlined, its loss contributes to many women's feelings of alienation and otherness. Such a situation confirms the need to look at a larger social scale. My interviewees generally did not live in marginalized areas as those studied by De Martino 1982[1959]; De Martino 2000[1958]) and Honkasalo (2009, 2015). However, their crisis of the presence could be positioned in a broader context of hyper-capitalism.

Together with the physical problems caused by the disease, the difficulty of finding a new place in society opens a crisis of the presence in which the loss of one's place in the world brings disorientation and existential anxiety.

## Conclusions

I have explored the material and moral uncertainties that characterize the experiences and subjectivities of women with MBC. The availability of new drugs for MBC and the possibility of enrollment in clinical trials creates complex therapeutic landscapes that are often challenging to navigate. Many patients live with uncertainty about their future; this uncertainty impedes repairing the disruption provoked by the illness that affects their working, social, and family lives.

The women with MBC have done practical and emotional work after their diagnosis, but such work is still not sufficient to bridge the fracture between the present and a future that is too uncertain and variable to be foreseen. Moreover, the work that the patients do encounters several barriers. Some of these barriers are intrinsic to the uncertain biomedical response to the illness, but this uncertainty is exacerbated by the social context in which the patients live. Kathy did not only have to deal with the uncertainty of therapies, but also with the moral uncertainty of having no access to a drug and the linked economic difficulties (although these were overcome in her case) when she decided to fund it herself. Moreover, for other women, both in the UK and in France, the illness restricted their professional, personal and social lives. The disruption deriving from these uncertainties not only limited the patients' bodies, but also challenged their capacity to plan a future and their place in the world. The crisis of the presence offers a heuristic capacity for considering the disorientation linked to this loss. De Martino 1982[1959], 2000[1958]) underscored how the loss of presence can be linked to critical moments of one's existence, but also how unfavorable economic and social relations can intensify the crises and the loss of presence. The crisis of the presence locates the anguish and disorientation brought by the diagnosis in a collective dimension of the materiality of the hyper-capitalistic society in which we live, rather than confining it to the individual dimension. Along with the uncertainty of its developments, MBC introduces bodily limitations that diminish the ability of the patients to conduct an active life. However, the crises are a direct consequence of the ableism of a hypercapitalist society that marginalizes those who cannot keep up with its rhythms, rather than making the rhythms more accessible. Women with MBC are painfully aware of their situation, and their agency is cut not by personal inabilities to react to the illness (coping) or to find new forms of living with the illness (adjustment). Such pacified registers point to personal solutions that are doomed to fail in this context. The situation invites further exploration of the social and political dimensions of illness experiences and the search for a recomposition of the disruption, not in the individual and personal dimension but in the collective and political dimension. This calls for a profound revision of how we consider illness, disabilities and individuals beyond their productive roles. It is only in a collective and historic dimension that a recomposition of the crisis and a restoration of the presence, albeit partial, is possible for those living with illness or disabilities.

## References

[R1] Abecassis P, Coutinet N (2017). Politique d’austérité et politique du médicament en France et au Royaume-Uni : Une analyse de leurs répercussions sur le modèle de production pharmaceutique. La Revue de l'Ires.

[R2] Andre F, Slimane K, Bachelot T, Dunant A, Namer M, Barrelier A, Kabbaj O (2004). Breast cancer with synchronous metastases: Trends in survival during a 14-year period. Journal of Clinical Oncology.

[R3] Bell K, Ristovski-Slijepcevic S (2011). Metastatic cancer and mothering: Being a mother in the face of a contracted future. Medical Anthropology.

[R4] Beneduce R (2005). Come curano le culture? Appunti sul guarire e l'efficacia simbolica, a partire da Ernesto de Martino. Rivista Sperimentale di Freniatria.

[R5] Biehl J, Locke P, Biehl J, Locke P (2017). Unfinished: The Anthropology of Becoming.

[R6] Brown P, de Graaf S (2013). Considering a future which may not exist: The construction of time and expectations amidst advanced-stage cancer. Health Risk & Society.

[R7] Burawoy M, Burawoy M, Burton A, Ferguson AA, Fox KJ, Gamson J, Gartrell N, Hurst L (1991). Ethnography Unbound: Power and Resistance in the Modern Metropolis.

[R8] Bury M (1982). Chronic illness as biographical disruption. Sociology of Health and Illness.

[R9] Bury M, Scambler G, Scambler S (2010). New Directions in the Sociology of Chronic and Disabling Conditions: Assaults on the Lifeworld.

[R10] Charmaz K (1983). Loss of self: A fundamental form of suffering in the chronically ill. Sociology of Health and Illness.

[R11] Crocetti L, De Baere T, Leoncini R (2010). Quality improvement guidelines for radiofrequency ablation of liver tumours. CardioVascular and Interventional Radiology.

[R12] De Martino E (1973). [1948] Il Mondo Magico.

[R13] De Martino E (1982). [1959] Sud E Magia.

[R14] De Martino E (2000). [1958] Morte e pianto rituale. Dal lamento funebre antico al pianto di Maria.

[R15] Delvecchio Good M-J (2001). The biotechnical embrace. Culture, Medicine and Psychiatry.

[R16] Garro LC, Mattingly C, Garro C, Mattingly LC (2000). Narrative and the Cultural Construction of Illness and Healing.

[R17] Gibbon S (2006). Nurturing women and the BRCA genes: Gender, activism, and the paradox of health awareness. Anthropology & Medicine.

[R18] Gibbon S (2007). Breast Cancer Genes and the Gendering of Knowledge: Science and Citizenship in the Cultural Context of the “New” Genetics.

[R19] Gobbini E, Ezzalfani M, Dieras V, Bachelot T, Brain E, Debled M, Jacot W (2018). Time trends of overall survival among metastatic breast cancer patients in the real-life ESME cohort. European Journal of Cancer.

[R20] Goodley D (2014). Dis/ability Studies: Theorising Disablism and Ableism.

[R21] Honkasalo M-L (2009). Grips and ties: Agency, uncertainty, and the problem of suffering in North Karelia. Medical Anthropology Quarterly.

[R22] Honkasalo M-L (2015). If the Mother of God does not listen: Women’s contested agency and the lived meaning of the Orthodox religion in North Karelia. Journal of American Folklore.

[R23] Jain SL (2013). Malignant: How Cancer Becomes Us.

[R24] Jordan VC (2006). Tamoxifen (ICI46,474) as a targeted therapy to treat and prevent breast cancer. British Journal of Pharmacology.

[R25] Keating P, Cambrosio A, Nelson NC (2016). “Triple negative breast cancer”: Translational research and the (re) assembling of diseases in post-genomic medicine. Studies in History and Philosophy of Biological and Biomedical Sciences.

[R26] Kenne Sarenmalm EK, Thorén-Jönsson A-L, Gaston-Johansson F, Öhlén J (2009). Making sense of living under the shadow of death: Adjusting to a recurrent breast cancer illness. Qualitative Health Research.

[R27] Klein R (2013). The New Politics of the NHS: From Creation to Reinvention.

[R28] Lewis S, Willis K, Yee J, Kilbreath S (2016). Living well? Strategies used by women living with metastatic breast cancer. Qualitative Health Research.

[R29] Löwy I (2007). Breast cancer and the “materiality of risk”: The rise of morphological prediction. Bulletin of the History of Medicine.

[R30] Manderson L, Warren N (2016). “Just one thing after another”: Recursive cascades and chronic conditions. Medical Anthropology Quarterly.

[R31] Mariotto AB, Etzioni R, Hurlbert M, Penberthy L, Mayer M (2017). Estimation of the number of women living with metastatic breast cancer in the United States. Cancer Epidemiology, Biomarkers & Prevention.

[R32] Oliver M, Barnes C, Mereer G (1996). Exploring the Divide: Illness and Disability.

[R33] Olson RE (2011). Managing hope, denial or temporal anomie? Informal cancer carers’ accounts of spouses’ cancer diagnoses. Social Science & Medicine.

[R34] Pandolfi M (1990). Boundaries inside the body: Women’s suffering in southern peasant Italy. Culture, Medicine and Psychiatry.

[R35] Pandolfi M (1993). Le self, le corps, la « crise de la présence ». Anthropologie et Sociétés.

[R36] Peláez-Ballestas I, Pérez-Taylor R, Aceves-Avila JF, Burgos-Vargas R (2013). ‘Not-belonging’: Illness narratives of Mexican patients with ankylosing spondylitis. Medical Anthropology.

[R37] Premkumar A, Kerns J, Huchko MJ (2020). “A résumé for the baby”: Biosocial precarity and care of substance-using, pregnant women in San Francisco. Culture, Medicine, and Psychiatry.

[R38] Sundquist M, Brudin L, Tejler G (2017). Improved survival in metastatic breast cancer 1985—2016. The Breast.

[R39] Timmermann C (2013). A History of Lung Cancer: The Recalcitrant Disease.

[R40] Trusson D, Pilnick A, Roy S (2016). A new normal?: Women’s experiences of biographical disruption and liminality following treatment for early stage breast cancer. Social Science & Medicine.

[R41] West J, Oldfather P (1995). Pooled case comparison: An innovation for cross-case study. Qualitative Inquiry.

[R42] Williams SJ (2000). Chronic illness as biographical disruption or biographical disruption as chronic illness? Reflections on a core concept. Sociology of Health & Illness.

